# Reducing behavioral health symptoms by addressing minority stressors in LGBTQ adolescents: a randomized controlled trial of Proud & Empowered

**DOI:** 10.1186/s12889-021-12357-5

**Published:** 2021-12-23

**Authors:** Jeremy T. Goldbach, Harmony Rhoades, Mary Rose Mamey, John Senese, Peter Karys, Flavio F. Marsiglia

**Affiliations:** 1 The Brown School, Washington University in St. Louis, 1 Brookings Dr, MO 63130 St. Louis, USA; 2 Suzanne Dworak-Peck School of Social Work, University of Southern California, 669 W. 34th St., MRF Bldg, CA 90089-0411 Los Angeles, USA; 3 The LGBT Community Center, 208 W. 13th St, 10011 New York, NY USA; 4 School of Social Work, Arizona State University, 411 N. Central Ave., Suite 720, 85004 Phoenix, AZ USA

**Keywords:** LGBT, Adolescent, Mental health, Intervention, Randomized controlled trial

## Abstract

**Background:**

Minority stress may lead to poorer mental health for sexual and gender minority adolescents, yet no interventions have been tested through an RCT to address these concerns.

**Methods:**

We report on an RCT of an intervention—Proud & Empowered—with four high schools. Measures assess the intervention’s impact on mental health symptoms.

**Results:**

Compared to the control, participants in the treatment condition reported significant differences in minority stress, anxiety, and depressive symptoms. Moderation analyses showed that the intervention significantly moderated the relationship between minority stress and PTSD (*b* = -1.28, *p* = .032), depression (*b* = -0.79, *p* = .023), and suicidality (*b* = 0.14, *p* = .012) symptoms; those in the intervention condition had mitigated relationships between measures of stress and health outcomes compared to those in the control condition.

**Conclusions:**

Results suggest that Proud & Empowered help reduce mental health symptoms and exposure to minority stressors and build coping strategies.

**Trial Registration:**

The intervention was registered on clinicaltrials.gov on August 1, 2019 under Trial #NCT04041414.

## Background

Sexual and gender minority adolescents (SGMA; i.e., youth who identify as something other than heterosexual and those with a gender identity that does not align with their assigned sex at birth) endure numerous behavioral health symptoms and disparities when compared to their heterosexual, cisgender peers. These disparities include higher rates of internalizing psychopathology [1], such as depression [2], anxiety [3], self-harm [4], and posttraumatic stress disorder (PTSD) symptomology [5], and externalizing behaviors such as substance use [6, 7] and suicide attempt and completion [8].

Minority stress theory explains these mental health disparities by suggesting that discrimination, violence, and victimization drives experiences of chronic minority stress [9, 10]. The long-term nature of these stress experiences place SGMA, aged 12–25 years, at higher risk of mental health conditions [11–13]. Evidence for this relationship between minority stress and behavioral health [11, 12] is clear, and displayed through numerous cross-sectional studies, including experiences of violent victimization [14], homophobic bullying [15], and family rejection in adolescence [11, 12, 16]. Similarly, studies have identified sexual minority-specific victimization [17] and stress experiences [10, 18] as mediating the relationships between sexual identity status and depression, PTSD, and suicidality [18–20]. These studies come together to provide robust evidence that improving youths’ ability to cope with minority stress is an important piece to improving mental health outcomes among SGMA, and that direct interventions are a means to fill this gap.

Despite a significant need to be addressed among SGMA, it has been noted that there are “no determinative studies, such as randomized control trials, of the efficacy and effectiveness of school based interventions [for SGMA]” (p.1767) [21], and the one U.S. based intervention with substantial quasi-experimental (the Family Acceptance Project [22]) relies heavily, on family participation. The challenge here is that many SGMA report not disclosing their sexual or gender identity to their family for fear of rejection [23], and those who do disclose their identity, often report a lack of family support [24, 25]. Another set of interventions developed in Canada, including Affirmative Supportive Safe and Empowering Talk and an affirmative behavioral coping skills group intervention [26, 27], as well as a Western Canadian media-based intervention to reduce bullying [28] have also found support, but their efficacy has yet to be established through randomized control designs in the literature. Thus, interventions that have a strong theoretical foundation, follow the National Institutes of Health’s gold-standard phase model of intervention development and testing, and rely solely on individual choice without family involvement are an essential but missing part of psychoeducational work needed to help SGMA cope with minority stress [5]. To address this gap in the literature, we developed a novel program, Proud & Empowered (P&E), to be delivered in school or community-based settings.

### Development of Proud & Empowered

The P&E intervention was named through a democratic vote by youth at LGBT centers after multiple rounds of development, implementation, and revision that followed the NIH Intervention Stage Model for Behavioral Intervention Development [29]. The 10-session small group intervention was supported by studies from 2012 to early 2020, most notably the development of a comprehensive minority stress measure for adolescents, the Sexual Minority Adolescent Stress Inventory (SMASI) [30, 31]. Although more detailed information can be found elsewhere [31], the SMASI emerged from a multiphase mixed-methods study including key informant interviews and focus groups [5]; life history calendar interviews [32] with 52 racially, ethnically, and gender diverse SGMA aged 14–17; a modified Delphi process [33] with an advisory panel of six experts in SGMA, minority stress theory, and psychometric development; and a rigorous validation process with adolescents (*N* = 346), including factor analytic and item response theory approaches [31]. The final SMASI measure found 10 conceptual domains: social marginalization, family rejection, internalized homonegativity, identity management, homonegative climate, intersectionality, negative disclosure experiences, religion, negative expectancies, and homonegative communication.

Considering the correlation between high stress in each domain and poorer health, we posited that ameliorating these stressors for SGMA would improve their health outcomes. Thus, the SMASI domains formed the basis for P&E’s 10 intervention sessions. Through an iterative process, consensus was reached that the intervention should include content on (a) stress and coping [9]; (b) disclosure decision-making [34]; (c) family; (d) school-related stress and resilience [35]; (e) peers and friendship [36]; (f) safety in relationships; (g) spirituality, faith, and religion [37]; (h) race, ethnicity, and social justice; (i) the LGBT community and history; and (j) intersections of health, substance use, HIV, and the medical system [38]. These aligned closely with the 10 SMASI domains and were agreed to by our research team, LGBT youth center staff, and the group of youth who participated in focus groups.

As intervention content was built, we also focused on understanding what strategies may be useful in coping for SGMA. Relying on the most commonly cited model of coping presented by Compas and colleagues [39], the study team identified both voluntary and involuntary physiological and emotional responses that may be related to SGMA minority stress, as well as engagement and disengagement strategies and coping resources with relevance to population health. In sum, we identified 16 population-specific coping resources that would further inform the intervention: for example, learning about the LGBTQ community, accessing factual historical information, or finding a supportive adult (ally). More detailed information on these novel coping resources is published elsewhere [5]. Finally, the intervention was pilot tested and further refined through a formative process with SGMA input, also published in a paper on the complete development process [40].

In sum, the final P&E intervention is a 10-session small group program for SGMA, with each session lasting approximately 45 min (one class period). A facilitator manual guides the curriculum; is used to monitor fidelity; and includes goals, learning objectives, activities, and a list of materials used for each session. Sessions rely on a mix of psychoeducation, didactic discussion, and interactive (e.g., role-play) activities. Following the previously described studies, the present article reports on a randomized controlled trial conducted at four public schools on the west coast of the United States to examine the efficacy of the P&E program. We hypothesized that youth who participated in P&E would (a) report significantly fewer minority stress experiences postintervention than those in the control group and (b) report significantly improved behavioral health symptomology (i.e., depression, PTSD, anxiety, and suicidality) compared to youth in the control group. We also examined whether participation in P&E moderated the relationship between minority stress and behavioral health symptoms to assess whether participation may improve youths’ ability to cope with minority stressors when they arise, because eliminating them is unfeasible.

## Methods

### Design

The randomized controlled trial was approved by the lead author’s institutional review board and was registered with clinicaltrials.gov. No major deviations occurred in the submitted protocol, and data collection for the intervention ended before COVID-19 resulted in school closures. The study was conducted with four schools in fall 2019 that collectively make up a unified school district in Southern California. The four schools’ range in size from 902 to 1,971 students each, have a combined enrollment of 5,706, and represent diverse racial and ethnic (e.g., 60% identify as Hispanic or Latinx and 16% as Black or African American) and income (e.g., 26% of families earn between $15,000 and $49,000 per year and 11% earn less than $15,000 per year) groups. All four schools had an active gender and sexuality alliance (GSA), but identified no other programming. The schools were randomly assigned to either an intervention or control condition. Youth in the intervention condition participated in the 10-week P&E program during one class period per week, whereas youth in the control condition attended school as usual during the same time period. Youth in both conditions completed measures at pretest and posttest, and measures were administered during the same week to both study conditions (pretest was one week prior to the first scheduled intervention session and posttest was 1 week after the last intervention session). We intended to complete a subsequent post-test measure, but restrictions due to the COVID-19 pandemic prevented this from occurring. Measures included demographic characteristics, our hypothesized mechanisms of change (i.e., minority stress), and mental health symptoms (i.e., depression, anxiety, PTSD, and suicidality). Participants received a $20 gift card for completing the measures at pretest and a $25 gift card at posttest.

#### Fidelity Monitoring

Fidelity monitoring was conducted using an approach developed in our prior pilot work [40] and was focused on adherence to curriculum, dosage, quality of service delivery, participant responsiveness, and program differentiation. [41] After each session, the facilitator and the liaison (co-facilitator) rated the objectives, content, and activities on fidelity, appropriateness, and participant receptiveness using an adherence checklist. Based on the core dimensions of the cultural adaptation model, [42] liaisons were asked to identify cognitive (comprehension), affective (cultural conflicts or motivation issues), developmental, and any other problems (e.g., environmental) with activities and rate fidelity to each session element (concepts, objectives, activities, instructions).

### Participants

Youth could participate in the P&E study if they (a) were students at the high school; (b) spoke English; (c) self-identified as LGBT or other non-heterosexual or cisgender identity; and (d) were willing and able to provide verbal assent. To identify potential participants, the study coordinator and school counselors made verbal presentations, distributed fliers, and had direct and confidential meetings to recruit youth to the study. Because LGBT youth represent a sensitive population and human subjects’ protection is complex (i.e., parents may not know their sexual or gender identity), we requested and received a waiver of parental consent by the institutional review board at the study team’s institution. Thus, potential youth participants received a detailed information sheet outlining the study goals, objectives, benefits, and possible risks and provided verbal assent or consent to participate (if they were 18 years old or turned 18 during the study).

Both school facilitators and student participants were informed that their school would be randomly selected to serve as either an intervention or control site. The district was recruited at the superintendent level, staff members at all schools were trained, students were administered pretests, and then schools were randomly assigned. We followed this process to help reduce the potential for bias in procedures.

### Procedures

#### Facilitator training

Although the intervention was led by a study team member, selected school staff members were trained to cofacilitate the curriculum to ensure the program could be readily administered by counselors, teachers, and other school personnel (e.g., social workers). Each school had up to two full-time staff members who received a 1-day training on the P&E curriculum overseen by the principal investigator and facilitated by the project coordinator. Most staff members were school counselors (*n* = 5), but also included teachers (*n* = 2) and other trained staff members. The training covered topics including: (a) minority stress among adolescents, (b) adolescent development and gender and sexual identity formation, (c) the NIH prevention principles, (d) P&E curriculum implementation, (e) fidelity monitoring processes, and (f) program outcomes and the research plan. Using previously established best practices [43], facilitators learned their roles in the project, identifying skills and concepts for each activity throughout the program. Facilitators received a $1,000 honorarium for their time in the training and participation throughout the school year.

#### Intervention condition

Participants were organized to meet weekly during their administration period (i.e., homeroom), as part of their regular school day, to participate in the intervention. Each group was led by the project coordinator and a school staff member who had been trained as a facilitator. Each session covered a different domain of minority stress identified and refined in our prior work (as previously described). A facilitator manual guided the curriculum, was used to monitor fidelity, and included goals, learning objectives, activities, and a list of materials used for each session.

#### Control condition

Although we considered whether an attention control [44] would be helpful in establishing intervention effects and reducing bias, we ultimately determined that attending classes as usual would be the most appropriate alternative activity, given that it would most closely match what youth would do if their school did not offer a focused intervention. Therefore, participants in the control condition did not participate in any unique activities; rather, they completed the surveys on the same timeline, and with the same incentive procedures, as the intervention group.

### Measures

#### Demographic characteristics

Demographic information was collected at both pre- and posttest and included gender, sex assigned at birth, age, grade, sexual orientation, and race. Gender categories included cisgender female, cisgender male, trans female or trans woman, trans male or trans man, and genderqueer or gender nonconforming. Sexual orientation had response options of gay, lesbian, bisexual, pansexual, asexual, and another sexual orientation (with the option to provide text). Participants could select all response options that applied on the race and ethnicity question, with options including non-Hispanic White, Black or African American, Latino or Hispanic, American Indian or Alaska Native, Native Hawaiian or other Pacific Islander, and another race and ethnicity with an open-ended text field. Other information collected included religion (both family and personal), language spoken (at home and with friends), whether the participant was currently working, and with whom the participant lives.

#### Minority stress

The SMASI features 54 items across 10 domains of minority stress for adolescents and demonstrates good reliability and validity in this population [30, 31]. Each statement reflects past-30-day thoughts, feelings, and situations a person may have experienced, with response options of 1 = *yes* and 0 = *no*. A decline-to-answer option was also provided. A total SMASI score was calculated by summing the 54 items. Mean imputation at the participant level was used for respondents missing fewer than seven items. Those missing seven or more items were removed from analysis. Scores for the 10 subscales were calculated using percentages; the number of endorsed (*yes*) responses were divided by the number of items a person actively responded to and multiplied by 100. The SMASI was collected at both time points.

#### Anxiety

Anxiety was measured using the 21-item Beck Anxiety Inventory [45]. Questions asked how much the person had been bothered by past-month symptoms, with response options of 0 (*not at all*), 1 (*mildly but it didn’t bother me much*), 2 (*moderately—it wasn’t pleasant at times*), and 3 (*severely—it bothered me a lot*). A sum score was created with a theoretical range between 0 and 63, with higher scores indicating higher levels of anxiety.

#### Depression

The Beck Depression Inventory II [46] is a list of 21 statements that describe how a person may have been feeling during the past 2 weeks, including sadness, loss of pleasure, and crying. Response options range from 0 to 3 and are unique to each question by catering to the specific feeling or behavior. Scores are calculated by summing all items and have a range between 0 and 63. Higher scores on the inventory demonstrate higher levels of depression.

#### PTSD

The PTSD Checklist for DSM-5 [47] was used to measure PTSD and assessed the extent to which the participant was bothered by 20 past-month experiences. Response options range from 0 (*not at all*) to 4 (*extremely*), with a possible sum score range between 0 and 80.

#### Suicidality

Suicidality was measured using the adapted Columbia-Suicide Severity Rating Scale [48] through five yes-or-no questions pertaining to the past 30 days. Worst-point severity is used to score the measure; a person’s last endorsed (*yes*) question is their score, with a range between 0 (no endorsed items) to 5 (a person endorsed the most severe question: “Have you thought about a specific plan [for example, having a time or place] to kill yourself?”). As a note, our human subjects plan included informing that if they endorsed suicidality, the facilitator would be informed confidentially (through Qualtrics skip logic) and that after class, they would be provided access to resources for dealing with suicidality, including an opportunity to speak privately with their school counselor to seek additional support. Eight participants endorsed suicidality at the pretest and received these resources.

#### Intervention

Each school was randomly assigned to the intervention or control condition. Thus, all students in a school received the same condition. Those in control schools were coded as 0, and those in intervention schools were coded as 1.

### Analysis

Descriptive information for demographics and outcome measures were first reported. Preliminary analyses used chi-square tests and one-way analyses of variance (ANOVA) to ensure demographic information and SMASI and mental health outcomes did not differ by group assignment (intervention vs. control) at Time 1. A two-by-two (group by time) repeated-measures ANOVA was used to understand the relationship between pre- and posttests across the intervention conditions for each outcome of minority stress and its subscales, and mental health symptoms of anxiety, depression, PTSD, and suicidality. These were designed to determine whether the intervention group differed in outcomes from the control group over time.

Subsequent analyses examined intervention as a moderator of the relationship between minority stress and mental health symptoms at Time 2, while controlling for mental health symptoms at Time 1. In each analysis, the mental health symptoms at Time 2 (dependent variable) were regressed onto the mental health symptoms at Time 1 (covariate), minority stress (independent variable), intervention (moderator), and the minority stress–intervention interaction term. Power analyses were conducted in G*Power 3.1.9.4 for both repeated-measures ANOVA and linear regressions with moderation. With 44 participants and α = 0.05, we were sufficiently powered (0.80) to detect an effect size of 0.12 for the repeated-measures ANOVA and an effect size of 0.18 for the moderation analysis. All analyses were conducted using SPSS version 25. Moderation analyses were conducted in the SPSS framework using PROCESS v. 3.4. [49].

## Results

**Fig. 1:**
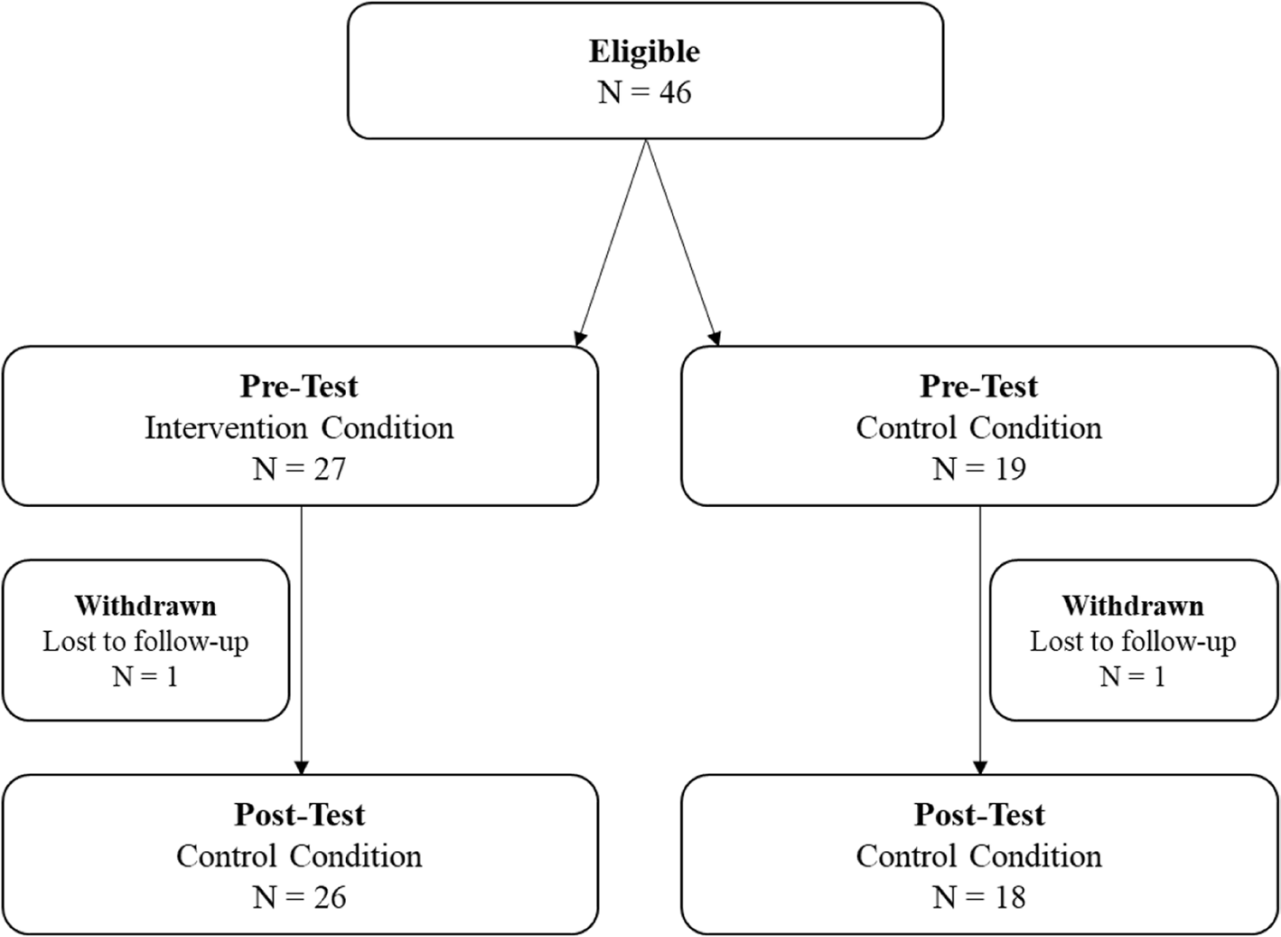
Consort Diagram

Of 46 students who actively participated in the study, two were removed for having not taken both the pre- and posttest (due to being absent at post-test). A consort diagram is provided below (Fig. 1).

Of the 44 participants retained for the study (95.6% retention rate), 26 students (59.1%) were in schools assigned to the intervention condition. Demographics are reported in Table 1. Chi-square tests were used to examine the relationship between group assignment and pretest demographic variables of interest. No significant differences were found between the intervention and control conditions for gender identity (χ^2^ = 7.36, *p* = .118), race (χ^2^ = 0.47, *p* = .828), age (χ^2^ = 6.04, *p* = .302), grade (χ^2^ = 6.64, *p* = .084), and sexual orientation (χ^2^ = 9.38, *p* = .153). Similarly, one-way ANOVAs were used to examine the relationship between group assignment and pretest outcome variables. No significant differences were found between minority stress total score (*F*[1] = 0.97, *p* = .330) or any of its subscales. Further, no significant differences were found between group assignment and mental health outcomes of anxiety (*F*[1] = 1.04, *p* = .315), PTSD (*F*[1] = 0.72, *p* = .402), depression (*F*[1] = 0.55, *p* = .474), or suicidality (*F*[1] = 0.09, *p* = .764).

**Table 1:** Frequencies of Primary Demographics of 44 Adolescents

	*n* (%)
*Group condition*	
Intervention	26 (59.1)
Control	18 (40.9)
*Gender at birth*	
Female	32 (72.7)
Male	12 (27.3)
*Gender identity*	
Female	27 (61.4)
Male	11 (25.0)
Trans female or trans woman	1 (2.3)
Trans male or trans man	2 (4.5)
Genderqueer or gender nonconforming	3 (6.8)
*Race* (all that apply)	
White	15 (33.1)
Black or African American	7 (15.9)
Latino or Hispanic	24 (54.5)
Asian	9 (20.5)
American Indian or Alaska Native	1 (2.3)
Native Hawaiian or Pacific Islander	1 (2.3)
*Age*	
13 years	4 (9.1)
14 years	7 (15.9)
15 years	11 (25.0)
16 years	12 (27.3)
17 years	9 (20.5)
18 years	1 (2.3)
*Grade*	
9th grade	8 (18.2)
10th grade	13 (29.5)
11th grade	9 (20.5)
12th grade	14 (31.8)
*Sexual orientation*	
Gay	8 (18.2)
Lesbian	6 (13.6)
Bisexual	18 (40.9)
Pansexual	5 (11.4)
Asexual	2 (4.5)
Queer	3 (6.8)
Straight	1 (2.3)
Decline to answer	1 (2.3)

Repeated-measures ANOVAs for minority stress and mental health outcomes were conducted to assess differences in intervention groups across the two time points. A significant interaction effect was found for SMASI subscales of internalized homonegativity (*F*[1] = 5.28, *p* = .028); the intervention condition decreased between Time 1 (*M* = 12.49, *SD* = 17.71) and Time 2 (*M* = 4.16, *SD* = 7.69), whereas the control condition increased between Time 1 (*M* = 3.30, *SD* = 8.56) and Time 2 (*M* = 7.69, *SD* = 16.09). Table 1 includes the means and standard deviations of the SMASI and its subscales. No significant findings were found for the SMASI total score, the other SMASI subscales, or the mental health outcomes. Means and standard deviations for SMASI and mental health outcomes can be found in Table 2.

**Table 2:** Past-30-day SMASI Scores and Health Outcomes of 44 Sexual Minority Youth

	Intervention	Control
	*M* (*SD*)	*M* (*SD*)
Total T1	8.44 (6.65)	7.22 (7.17)
Total T2	7.59 (7.13)	8.29 (7.68)
Identity management T1	19.05 (29.00)	15.38 (29.24)
Identity management T2	14.29 (22.54)	17.95 (25.88)
Negative expectancies T1	13.64 (28.47)	12.82 (21.68)
Negative expectancies T2	21.21 (31.78)	23.08 (34.39)
Negative disclosure experiences T1	11.82 (21.96)	12.31 (22.42)
Negative disclosure experiences T2	8.18 (21.08)	12.31 (23.86)
Family rejection T1	19.67 (30.62)	14.90 (25.44)
Family rejection T2	17.44 (28.67)	14.34 (23.02)
Internalized homonegativity T1	12.49 (17.71)	3.30 (8.56)
Internalized homonegativity T2	4.16 (7.04)	7.69 (16.09)
Homonegative communication T1	46.36 (32.88)	41.15 (25.67)
Homonegative communication T2	42.50 (30.15)	47.69 (26.51)
Homonegative climate T1	11.36 (16.77)	5.77 (20.80)
Homonegative climate T2	17.05 (28.23)	13.46 (26.25)
Social marginalization T1	1.70 (4.39)	1.92 (4.69)
Social marginalization T2	2.44 (6.90)	2.88 (5.48)
Intersectionality T1	10.61 (26.00)	28.21 (40.47)
Intersectionality T2	10.61 (23.87)	17.95 (32.25)
Religion T1	11.14 (16.18)	12.69 (17.63)
Religion T2	13.18 (19.85)	13.85 (18.95)
Anxiety T1	22.08 (14.44)	26.89 (16.75)
Anxiety T2	19.56 (16.08)	27.39 (14.14)
PTSD PCL T1	21.81 (18.30)	26.78 (20.34)
PTSD PCL T2	24.40 (19.08)	29.28 (17.32)
Depression (Beck II) T1	15.40 (11.54)	17.81 (9.56)
Depression (Beck II) T2	15.68 (11.96)	18.65 (12.77)
Suicide T1	0.63 (1.31)	0.50 (1.34)
Suicide T2	0.71 (1.46)	0.61 (1.24)
PTSD 6-item T1	13.42 (6.00)	15.24 (7.15)
PTSD 6-item T2	14.68 (6.30)	16.50 (5.51)

Analyses were further conducted to examine whether the intervention group moderated the association between minority stress and mental health symptoms. Minority stress had a statistically significant interaction with PTSD (*b* = -1.29, *p* = .032); for those in the intervention group, higher levels of minority stress were associated with lower levels of PTSD (*b* = -0.48, *p* = .234), and for those in the control group, higher levels of minority stress were associated with higher levels of PTSD (*b* = 0.81, *p* = .063). This significant interaction suggests that the relationship between minority stress and PTSD differed between groups. Results also demonstrated a significant moderation effect of the intervention between minority stress and depression (*b* = -0.79, *p* = .023). Higher levels of minority stress were associated with lower levels of depression for those in the intervention condition (*b* = -0.36, *p* = .131), whereas higher levels of minority stress were associated with higher levels of depression for those in the control condition (*b* = 0.43, *p* = .093). The intervention also significantly moderated the relationship between minority stress and suicidality (*b* = -0.14, *p* = .012); higher levels of minority stress were associated with lower levels of suicidality for the intervention condition (*b* = -0.07, *p* = .069), and higher levels of minority stress were associated with higher levels of suicidality for the control condition (*b* = 0.07, *p* = .087). Each of these findings show the intervention reduced the strength of the relationship between higher levels of minority stress and PTSD, depression, and suicidality when compared to the control condition. Table 3 reports these findings.

**Table 3:** Moderated Analyses with SMASI by Intervention Interaction

Predictors	Anxiety	
	*b*	*p*
SMASI	0.14	0.730
Intervention	-4.16	0.267
Anxiety (T1)	0.76	< 0.001
SMASI x intervention	-0.28	0.610
Predictors	PTSD	
	*b*	*p*
SMASI	0.81	0.063
Intervention	-2.49	0.514
PTSD (T1)	0.76	< 0.001
SMASI x intervention	-1.29	0.032
Intervention	-0.48	0.234
Control	0.81	0.063
Predictors	Depression	
	*b*	*p*
SMASI	0.43	0.093
Intervention	-1.24	0.587
Depression (T1)	0.96	< 0.001
SMASI x intervention	-0.79	0.023
Intervention	-0.36	0.131
Control	0.43	0.093
Predictors	Suicidality	
	*b*	*p*
SMASI	0.07	0.087
Intervention	-0.07	0.841
Suicidality (T1)	0.41	0.007
SMASI x intervention	-0.14	0.012
Intervention	-0.07	0.069
Control	0.07	0.087

## Discussion

This randomized controlled trial demonstrated evidence for the preliminary effectiveness of the P&E prevention intervention. Because SGMA experience numerous mental health disparities when compared to their heterosexual peers, implementing interventions like P&E may be crucial to promoting mental wellness and coping among these youth, who often manifest these experiences of minority stress as mental health symptomology throughout their lives [12, 30].

Supporting our first hypothesis, we found evidence that participation in the P&E intervention reduced minority stress among intervention participants. Even with a sample of fewer than 50 youth across intervention and control conditions, we found statistically significant decreases in minority stress experiences among youth in the intervention group, whereas experiences of minority stress increased among those in the control condition. This suggests the intervention is successful in decreasing minority stress through reframing the understanding and approach of stressors in the control of participants. However, further research is needed with larger samples, because it was difficult to see changes in subscales of our minority stress measure, given sampling and power challenges.

As we also expected based on literature linking minority stress experiences with mental health [11, 12], we found evidence suggesting that P&E reduces negative mental health symptoms (Hypothesis 2). Those in the intervention group reported decreased symptoms of anxiety and no change in depression symptoms, as compared to those in the control group, who saw no change in anxiety and an increase in depression symptoms. These findings suggest protective effects of the intervention for mental health symptoms among LGBT students. The knowledge youth gained through the stress-based psychoeducation aspect of P&E broadened their understanding of how minority stress plays a role in the symptoms they experience. This understanding may promote mindful identification and coping with symptoms of stress and anxiety, as found in other studies with adolescents [50]. Furthermore, youth gained peer support through group sharing of common experiences, which has been shown to be related to positive mental health and well-being among SGMA [51].

Finally, we found preliminary evidence that P&E moderated the relationship between minority stress experiences and mental health symptoms. Analyses identified significant interactions between minority stress experiences and the intervention condition, suggesting that youth in the intervention group were less likely than those in the control group to report elevated PTSD, depression, and suicidality in the face of minority stress experiences. Although we designed P&E to address many types of proximal experiences of minority stress (e.g., internalized homonegativity, identity management), we recognize that a small group intervention can only go so far to address distal stressors such as homonegative school climates and peer and family rejection. Given that these stressors would be difficult (or impossible) to eliminate with an individual-based intervention, they are likely to be experienced regardless of intervention participation. Thus, we were pleased to see that P&E appears to provide participants with improved coping skills that may help them in the face of these distal stress experiences. Our findings provide evidence that the intervention moderated the relationship between minority stress and mental health symptoms. That is, even in cases where the intervention may not reduce certain aspects of minority stress, it nevertheless provided youth with tools to cope with those experiences and help prevent subsequent exposure.

Our study is not without limitations. First, we recruited a relatively small sample size in a limited number of schools in one geographic area of the United States. Thus, although some of our findings trended toward significance, we could not detect changes in several of our hypothesized outcome measures, conduct subgroup tests of difference to examine differential impacts (e.g., by race, ethnicity, gender identity), nor look at how school-level structural factors may affect student outcomes. This is a critical challenge of the present study and a subsequent study, with a larger sampling frame, is needed to further understand the relationship between program participation, minority-related stress and behavioral health symptomology. Additionally, recruitment of hard-to-reach youth proved to be successful in this study; nonetheless, we recognize that some youth who are not out to their peers may have been reluctant to participate, and that we may find differing rates of participation in more rural or underserved areas of the United States. Future studies of P&E should be conducted with larger samples to determine its effectiveness in more geographically diverse areas.

## Conclusions

Despite limitations, this study provides preliminary evidence for the effectiveness of P&E. The significant findings, despite the relatively small sample size, suggest that the intervention holds promise for reducing experiences of minority stress and mental health symptoms and provides youth with ways to cope with experiences of minority stress. Various reports have identified SGMA as at risk of developing chronic psychopathology and implementing preventive interventions such as P&E early may be crucial for preventing significant health disparities from developing in the first place.

## Data Availability

The data can be made available with appropriate IRB approvals by emailing the lead author.

## Abbreviations

ANOVA: Analyses of Variance
COVID-19: Coronavirus Disease 2019
DSM-5: Diagnostic and Statistical Manual of Mental Disorders, Fifth Edition
HIV: Human Immunodeficiency Virus
LGBT: Lesbian, Gay, Bisexual, and Transgender
LGBTQ: Lesbian, Gay, Bisexual, Transgender and Queer or Questioning
NIH: National Institutes of Health
P&E: Proud & Empowered
PTSD: Post-Traumatic Stress Disorder
RCT: Randomized Controlled Trial
SGMA: Sexual and Gender Minority Adolescents
SMASI: Sexual Minority Adolescent Stress Inventory
